# Live Sextuplet Birth After Ovulation Induction in a Patient With Secondary Infertility: A Case Report

**DOI:** 10.7759/cureus.113500

**Published:** 2026-07-28

**Authors:** Alla Abdelgader, Ibrahim Daaoud, Azza Mohamed Ali Dawelbait, Baharelden Abuobida, Wafa Mahgoub, Aalaa Almuazel, Alsiddig Yousef Modwey, Amjed Elkheir, Fath Elrahman Elrasheed, Awadalla Abdelwahid, Abdelrazig E Abdelbari, Mohannad Mohamed, Duha Mohammed

**Affiliations:** 1 Obstetrics and Gynecology, Tadawi Hospital, Khamis Mushait, SAU; 2 Obstetrics and Gynaecology, Faculty of Medicine, Alneelein University, Khartoum, SDN; 3 Obestetric and Gynacology, Portiuncula University Hospital, Galway, IRL; 4 Consultant of Obstetrics and Gynecology, Bader Aljanoub Hospital, Najran, SAU; 5 Obstetrics and Gynecology, Dr. Suliman Alhabib Medical Group, Women Health Hospital, Riyadh, SAU; 6 Obstetrics and Gynecology, Specialized Medical Company Hospital [SMC1], Riyadh, SAU; 7 Obstetrics and Gynaecology, Dahran Aljnoub General Hospital, Dahran Aljnoub, SAU; 8 Obstetrics and Gynecology, Prince Sultan Military Medical City, Riyadh, SAU; 9 Obstetrics and Gynaecology, Najran Unversity, Najran, SAU; 10 Obstetrics and Gynaecology, Alneelain University, Khartoum, SDN; 11 Family Medicine Specialist, The Executive Administration for Healthcare Delivery, Najran, SAU; 12 Obstetrics and Gynecology, Almogtarbeen University, Khartoum, SDN; 13 Obstetrics and Gynecology, Abha International Private Hospital, Abha, SAU

**Keywords:** cervical cerclage, clomiphene citrate, high-order multiple pregnancy, ovulation induction, preterm birth, unexplained secondary infertility

## Abstract

High-order multiple pregnancy is an unusual but serious complication of ovulation induction and is associated with significant maternal, fetal, and neonatal risks. While ovulation induction has been commonly used in the management of unexplained infertility, multifetal gestation remains an important iatrogenic outcome despite careful monitoring. In this article, we report a rare case of high-order multiple pregnancy following the induction of clomiphene citrate in a woman with unexplained secondary infertility.

A 30-year-old para 1 female patient (eight years long) presented to Tadawi Medical Hospital, Khamis Mushait, Saudi Arabia, seeking pregnancy for secondary infertility. An infertility study confirmed normal hormonal status, bilateral tubal patency on hysterosalpingography, and normal semen analysis of her husband, and a diagnosis of unexplained secondary infertility was made. At the start of the stage, she underwent routine cycle monitoring using timing for intercourse and received folic acid, vitamins, and L-arginine. Induction of ovulation was accomplished by clomiphene citrate 50 mg daily for five days. On day 12, she had mild ovarian hyperstimulation, managed lightly. Initial early pregnancy evaluation showed markedly increased beta-human chorionic gonadotropin levels. Ultrasound revealed five gestational sacs that each harbored a viable fetal pole, pointing towards high-order multifetal gestation. The patient received close antenatal follow-up every two weeks. Fetal reduction was recommended due to the high maternal and perinatal risks; however, she declined. Cervical cerclage at 14 weeks gestation with Mersilene was performed because of the increased risk of cervical insufficiency. She was kept on progesterone support and was placed on dexamethasone for fetal lung maturation at 24 weeks. Serial ultrasounds revealed continuous viability of all fetuses and no gross structural anomalies. Although elective delivery was planned at 30-32 weeks, she developed preterm labor at 28 weeks and underwent cesarean section. Each neonate was born alive and placed in the neonatal intensive care unit for approximately five weeks.

A rare situation with an extremely serious management challenge of high-order multiple pregnancy, including the complications post-ovulation induction and management challenges that this case presents. To facilitate these outcomes, it is critical that intensive monitoring, timely intervention, complex management, multidisciplinary effort, as well as neonatal support occur.

## Introduction

Millions of couples worldwide experience infertility, and unexplained infertility is a diagnosis of exclusion made after confirmation of ovulation, tubal patency, and normal semen analysis [1,2]. Current guidelines recognize unexplained infertility as an important subgroup of infertility and recommend a stepwise, evidence-based management approach based on the patient’s age, prognosis, and duration of infertility [2].

For many women, particularly those with secondary infertility, ovulation induction combined with timed intercourse or intrauterine insemination is the most common treatment before proceeding to more advanced assisted reproductive technologies [2,3]. Although ovarian stimulation may improve the likelihood of conception, it also increases the risk of multifollicular development and iatrogenic multiple gestation, particularly with gonadotropin therapy or when cycle cancellation criteria are less stringent [3,4].

Current reproductive medicine guidelines identify the birth of a single healthy infant as the primary goal of infertility treatment, and preventing treatment-associated multifetal pregnancy remains a major priority [5,6]. Population-based data published in 2024 further indicate that although in vitro fertilization has reduced the incidence of multifetal pregnancy, continued efforts are needed to decrease multiple gestations associated with ovulation induction and intrauterine insemination [7].

High-order multiple pregnancy has become uncommon but remains associated with substantial maternal and neonatal morbidity. Multifetal gestation is associated with miscarriage, hypertensive disorders, anemia, fetal growth restriction, preterm premature rupture of membranes, preterm birth, low birth weight, early neonatal intensive care unit admission, and perinatal mortality [5,6]. These risks increase with the number of fetuses.

Preterm birth is the most significant complication of triplet and higher-order pregnancies, and preventive interventions have shown limited effectiveness in prolonging gestation [5]. Cervical shortening and cervical insufficiency present additional challenges because of marked uterine overdistension and the inherently high risk of very preterm delivery. Emerging evidence suggests that, in carefully selected women with multifetal pregnancies and a very short cervix, cervical cerclage may prolong pregnancy and improve selected neonatal outcomes when appropriately used, although the quality of the evidence remains low [8].

We report the unusual case of a live sextuplet birth following ovulation induction in a patient with unexplained secondary infertility. After cervical cerclage, all six live neonates were delivered at 28 weeks’ gestation. This case highlights the complexity of counseling, antenatal surveillance, and multidisciplinary management in high-order multiple pregnancy.

## Case presentation

A 30-year-old para 1 woman presented to the Obstetrics and Gynecology Clinic at Tadawi Medical Hospital, Khamis Mushait, Saudi Arabia, with an eight-year history of secondary infertility and a desire to conceive. An initial infertility evaluation revealed no identifiable abnormalities. Her hormonal profile was within normal physiological limits, and a hysterosalpingogram performed at Tadawi Medical Hospital demonstrated a normal uterine cavity, bilaterally patent fallopian tubes, and free bilateral peritoneal spill.

Her husband’s semen analysis was also normal. Based on these findings, she was diagnosed with unexplained secondary infertility. Initial conservative management consisted of cycle monitoring, including antral follicle count on day two and repeat follicular assessment on day 12, together with advice on timed intercourse for two cycles. She also received supportive therapy with vitamins, folic acid, and L-arginine.

Because pregnancy did not occur, ovulation induction was initiated with clomiphene citrate 50 mg orally once daily for five days. On day 12, she developed clinical and ultrasonographic findings consistent with mild ovarian hyperstimulation syndrome, which was managed conservatively with analgesia, adequate oral hydration, and continued timed intercourse.

**Figure 1:**
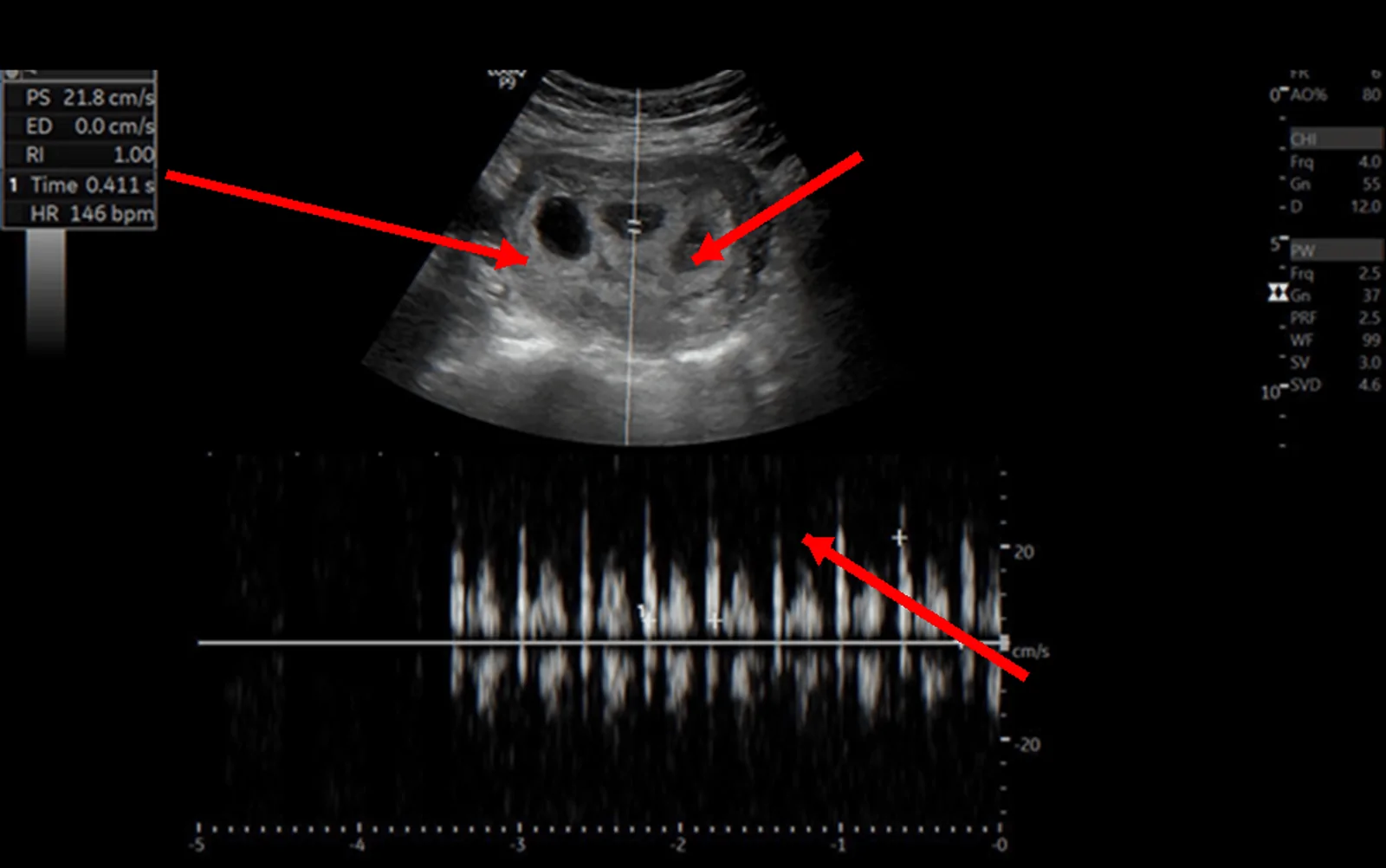
Early Ultrasound of High-Order Multiple Pregnancy Early first-trimester obstetric ultrasound with Doppler imaging demonstrating multiple intrauterine gestational sacs with fetal cardiac activity, consistent with a high-order multiple pregnancy. Red arrows indicate the gestational sacs and the Doppler fetal cardiac activity trace.

**Figure 2:**
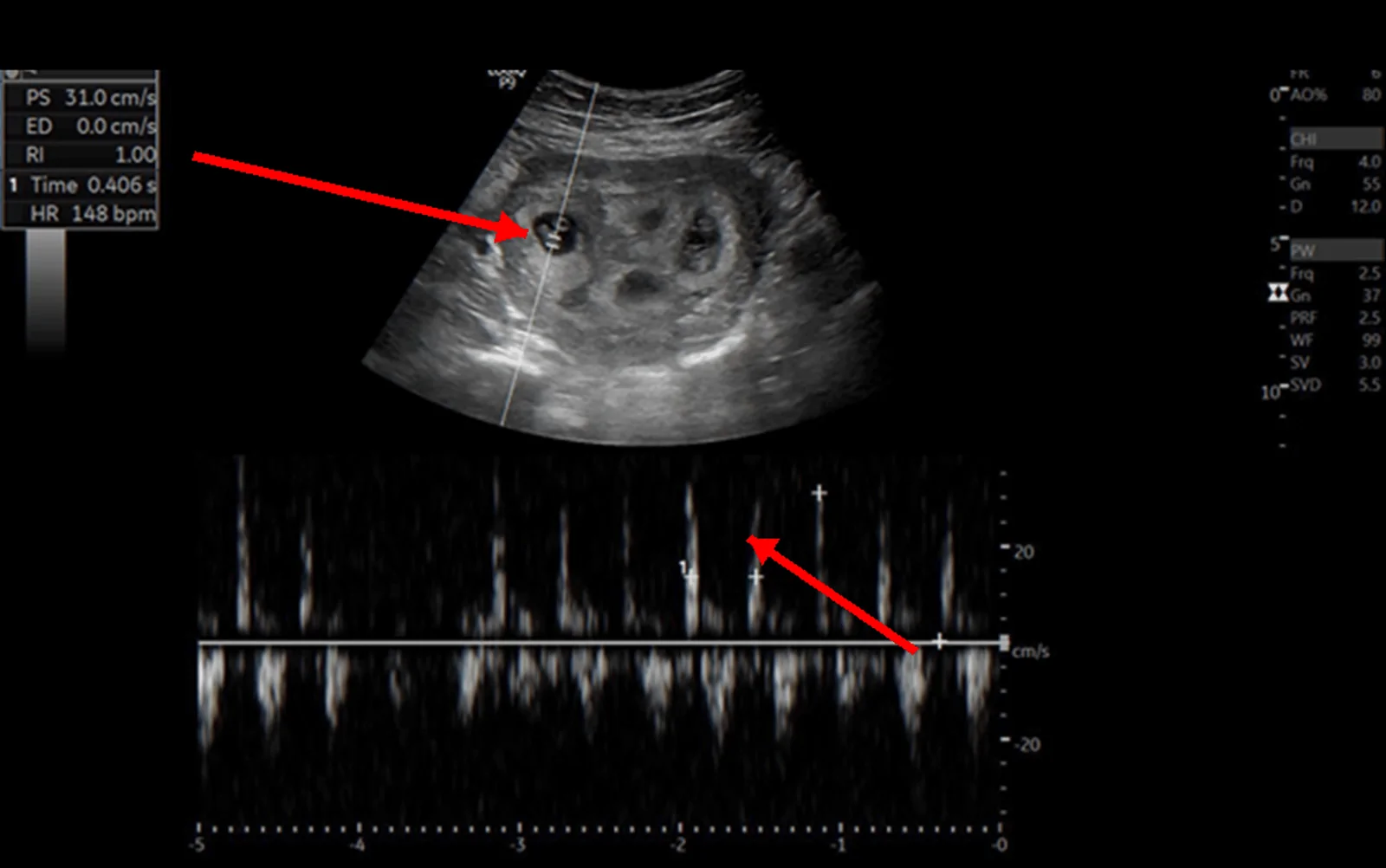
First-Trimester Fetal Cardiac Activity Follow-up first-trimester ultrasound with Doppler imaging showing viable fetal cardiac activity in one of the gestational sacs. Red arrows indicate the gestational sac/fetal pole and the Doppler cardiac activity waveform.

At approximately six weeks’ gestation, serum beta-human chorionic gonadotropin levels were markedly elevated. An obstetric ultrasound demonstrated a markedly enlarged uterus containing multiple gestational sacs with viable fetal poles, corresponding to approximately seven weeks and three days’ gestation (Figures 1, 2).

Bilateral multiple ovarian cysts were also identified, the largest measuring 5.5 cm in the right ovary and 4.5 cm in the left ovary, with a small amount of free fluid in the pouch of Douglas. The patient was followed in the antenatal clinic every two weeks.

She received extensive counseling regarding the substantial maternal and fetal risks associated with a high-order multiple pregnancy and was offered fetal reduction, which she declined. At 14 weeks’ gestation, she was admitted for cervical cerclage because of suspected cervical insufficiency.

**Figure 3:**
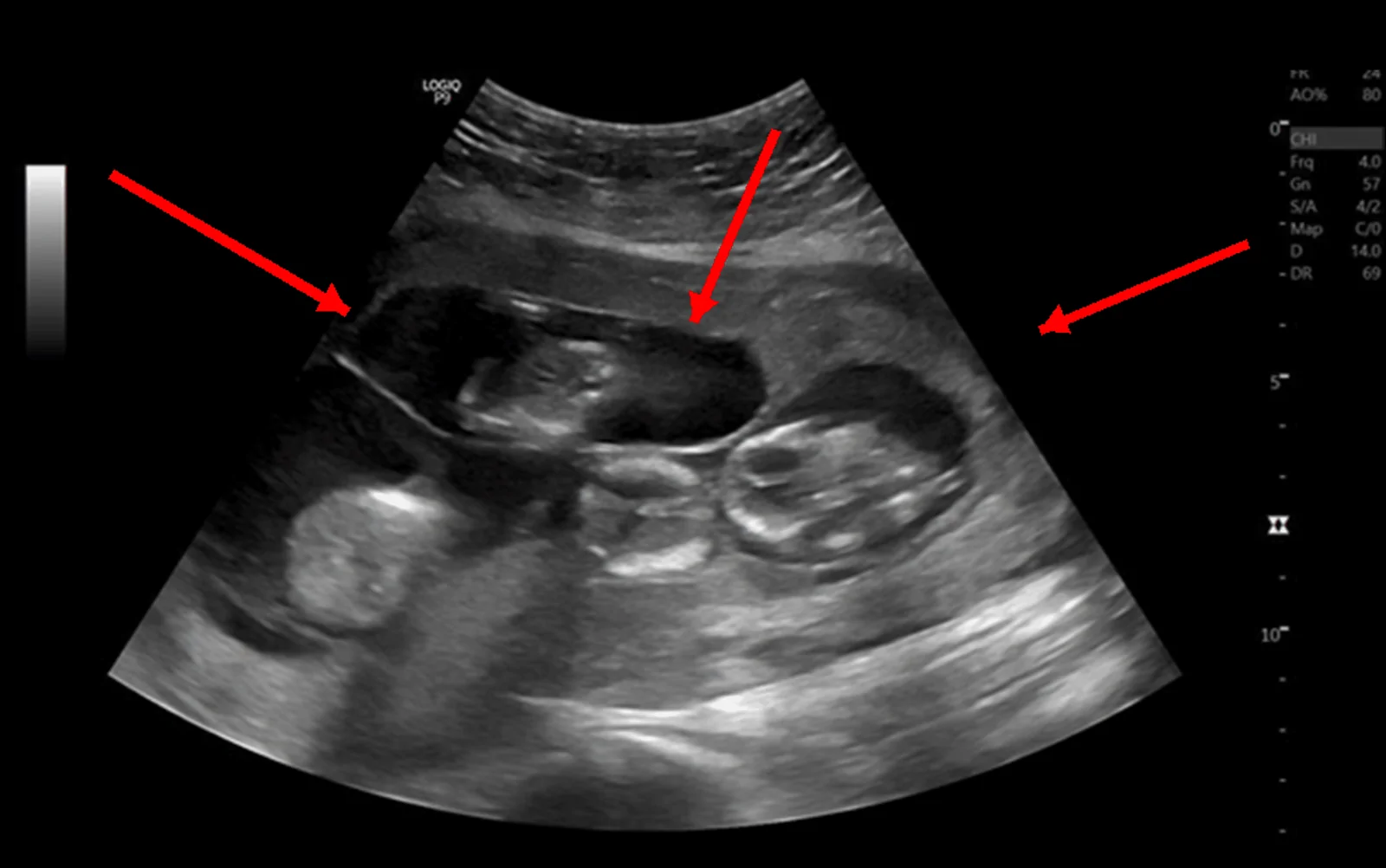
Multiple Viable Fetuses at 14–15 Weeks Ultrasound image obtained at approximately 14-15 weeks’ gestation demonstrating multiple viable intrauterine fetuses in a high-order multiple pregnancy. Red arrows indicate the fetal parts within separate gestational compartments.

**Figure 4:**
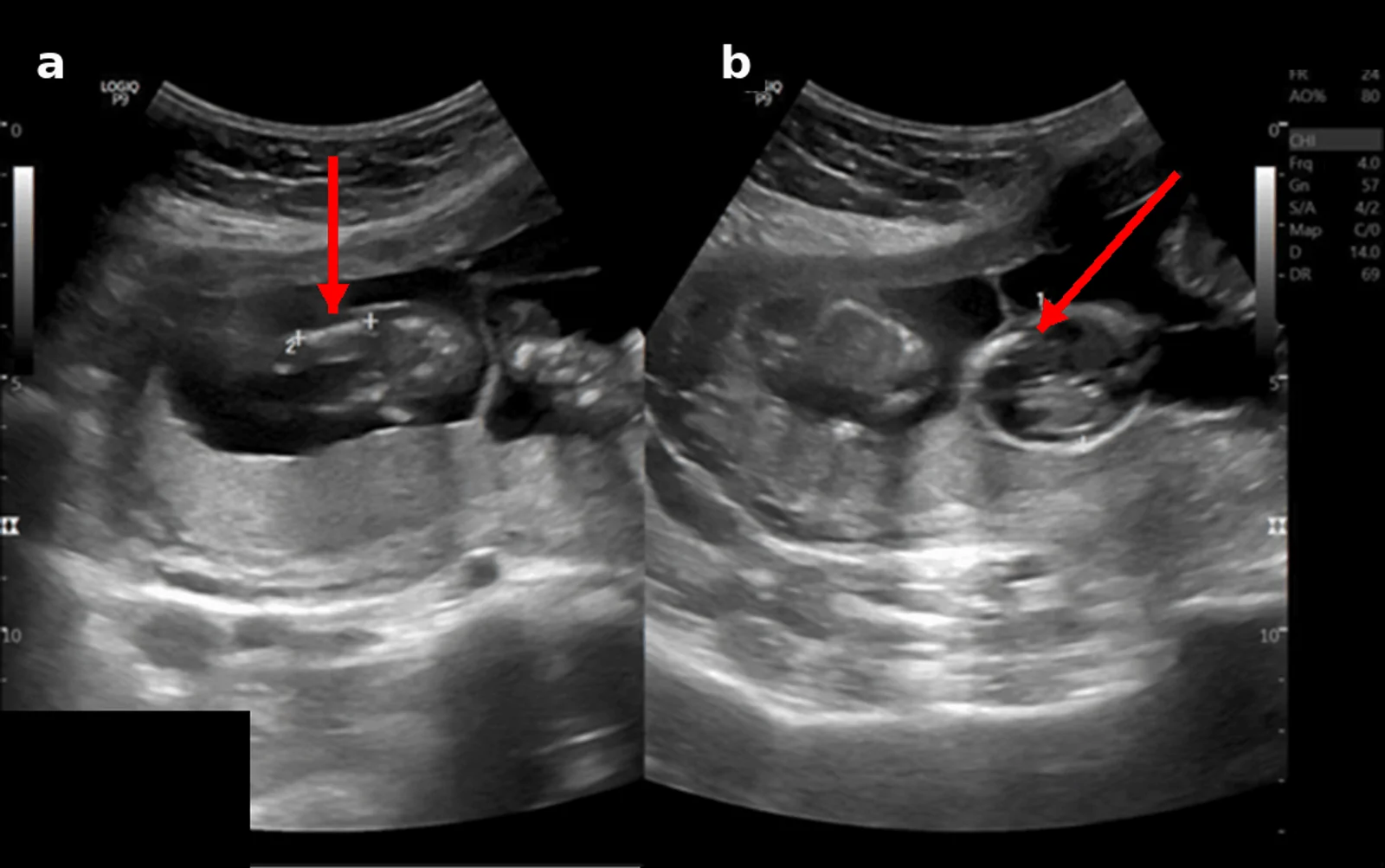
Fetal Biometric Assessment at 14 Weeks Obstetric ultrasound obtained at 14 weeks and five days’ gestation showing fetal biometric assessment. Panel (a) demonstrates femur length measurement, and panel (b) demonstrates cranial biometric assessment. Red arrows indicate the measurement areas of interest. Dates visible on the original scan have been obscured.

**Figure 5:**
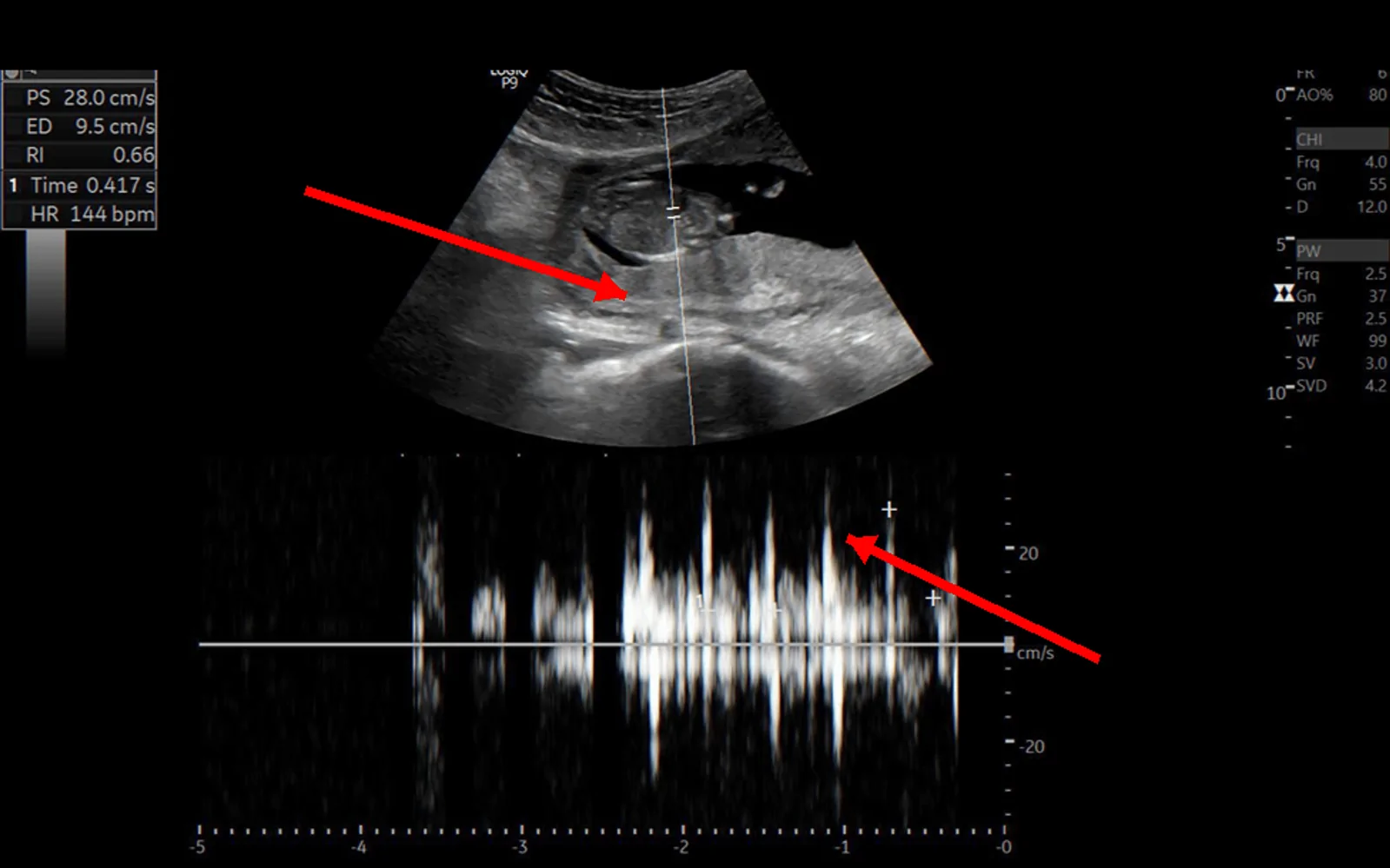
Doppler Assessment of Fetal Heart Rate Doppler ultrasound obtained at 14 weeks and 5 days’ gestation demonstrating fetal cardiac activity with a heart rate of approximately 144 beats per minute. Red arrows indicate the fetal cardiac region and Doppler waveform.

An ultrasound confirmed five viable intrauterine fetuses with a mean gestational age of 14 weeks and 5 days, multiple placentas, normal fetal movements and cardiac activity, and no gross congenital anomalies on routine examination (Figures 3, 4, 5). The internal cervical os measured 7 mm.

Cervical cerclage was performed using Mersilene suture material. She remained on Duphaston and Cyclogest 400 mg twice daily and received dexamethasone at 24 weeks’ gestation to promote fetal lung maturation.

**Figure 6:**
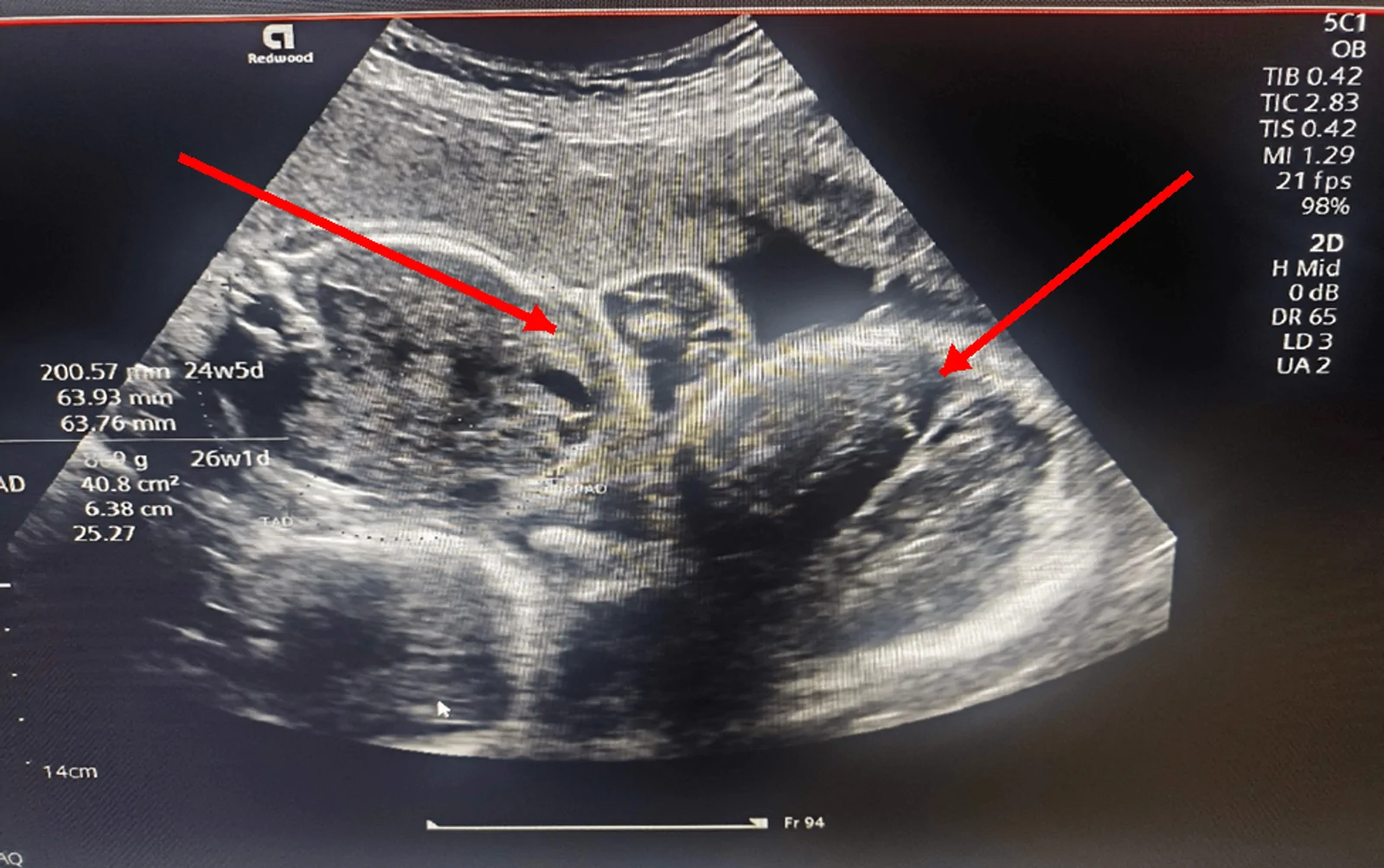
Second-Trimester Fetal Biometric Assessment Second-trimester ultrasound obtained at approximately 24-26 weeks’ gestation demonstrating an ongoing viable high-order multiple pregnancy with fetal biometric assessment. Red arrows indicate the fetal biometric region and the fetal body parts assessed during surveillance.

**Figure 7:**
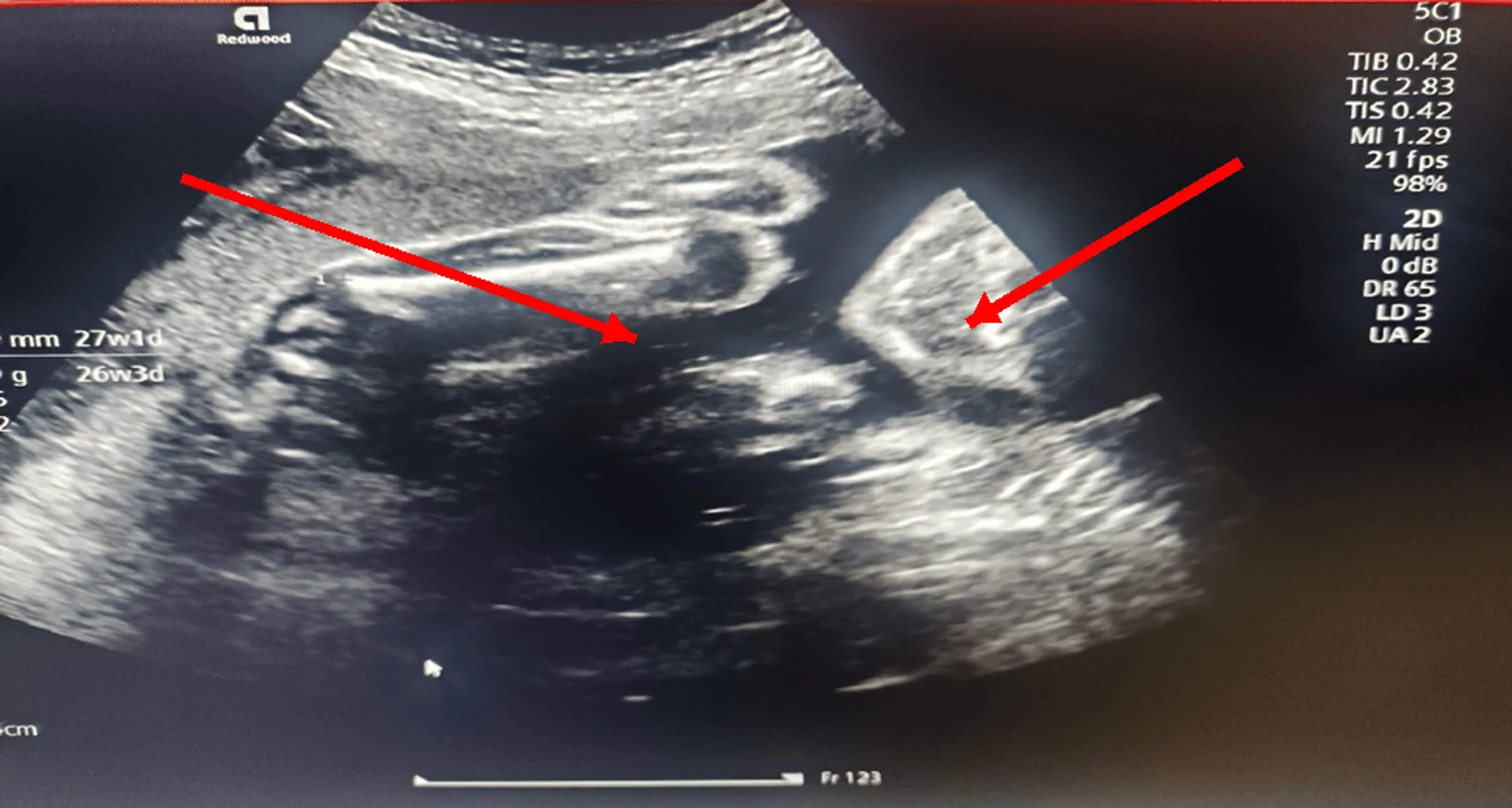
Fetal Growth Assessment at 26–27 Weeks Second-trimester ultrasound obtained at approximately 26-27 weeks’ gestation demonstrating fetal growth assessment and continued viability during antenatal surveillance. Red arrows indicate the fetal biometric region and fetal parts assessed during follow-up.

Subsequent antenatal surveillance demonstrated no gross fetal structural abnormalities, and glucose tolerance testing was normal. A later ultrasound showed viable intrauterine fetuses with estimated gestational ages of 26-27 weeks and estimated fetal weights of 780 g, 860 g, 1,000 g, 930 g, and 1,000 g (Figures 6, 7). Amniotic fluid volume was normal and clear, with no evidence of retroplacental collection.

Elective delivery was planned for 30-32 weeks’ gestation while maternal and fetal conditions remained stable. However, the patient went into preterm labor at 28 weeks’ gestation and underwent cesarean delivery with the pediatric team in attendance.

All six neonates were born alive and received care in the neonatal intensive care unit for approximately five weeks.

## Discussion

This case illustrates several important issues in the management of unexplained secondary infertility complicated by a high-order multiple gestation following ovulation induction. Although clomiphene citrate is widely used as an accessible first-line treatment, it still carries a risk of multiple pregnancy.

Current evidence indicates that the overall multiple pregnancy rate with contemporary single-agent clomiphene protocols is lower than historically reported. In contrast, high-order multiple pregnancies remain rare but represent a serious iatrogenic complication with significant maternal and neonatal consequences [9,10]. In this patient, conception occurred following monitored ovulation induction and timed intercourse, but the pregnancy progressed to sextuplets, demonstrating that even low-dose stimulation can occasionally produce an extreme ovarian response.

High-order multiple pregnancies are associated with substantial obstetric risks, including anemia, hypertensive disorders, preterm birth, operative delivery, and adverse perinatal outcomes. Observational studies consistently identify preterm birth as the leading complication, with neonatal survival depending largely on gestational age at delivery and access to neonatal intensive care [11]. This is consistent with the present case, in which delivery occurred at 28 weeks’ gestation and all six neonates required admission to the neonatal intensive care unit.

Recent reports of other high-order multifetal pregnancies indicate that successful neonatal survival is strongly associated with intensive antenatal surveillance, multidisciplinary delivery planning, and advanced neonatal care [12]. Counseling in these pregnancies is particularly challenging. Systematic reviews have shown that fetal reduction is associated with a higher gestational age at delivery, lower rates of preterm birth, and improved birth weight; however, decisions regarding fetal reduction are highly individualized and involve complex ethical considerations [13].

In the present case, fetal reduction was recommended because of the substantial maternal and neonatal risks, but the patient declined. Her decision required enhanced antenatal surveillance and ongoing counseling within a patient-centered model of care.

The role of cervical cerclage in multifetal gestation also remains controversial. Although the evidence is less robust than for singleton pregnancies, recent meta-analyses suggest that cerclage in carefully selected women with a very short cervix may reduce the risk of early preterm birth and improve neonatal outcomes [14]. Other recent reviews likewise support emergency or indication-based cerclage in selected cases while emphasizing that the available evidence remains limited and that counseling should be undertaken with caution [15,16]. In this case, prophylactic cerclage performed at 14 weeks’ gestation may have contributed to prolonging the pregnancy until 28 weeks.

Finally, this case highlights that neonatal survival is only one measure of success. High-order multiple pregnancies are strongly associated with extreme prematurity. Although survival rates have improved, very preterm infants remain at increased risk of long-term neurodevelopmental impairment and multisystem complications [13,17]. Accordingly, long-term pediatric follow-up is essential for all six infants.

Overall, this case demonstrates the rarity of sextuplet pregnancy following ovulation induction, the clinical and ethical challenges associated with its management, and the importance of careful ovarian stimulation protocols, close antenatal surveillance, multidisciplinary collaboration, and comprehensive neonatal care.

## Conclusions

This case demonstrates that extreme high-order multifetal pregnancy can occur even after low-dose ovulation induction with clomiphene citrate. Sextuplet pregnancy carries substantial maternal and perinatal risks, particularly cervical shortening, very preterm birth, low birth weight, operative delivery, and prolonged neonatal intensive care. In this patient, close antenatal surveillance, comprehensive counseling, cervical cerclage, progesterone support, antenatal corticosteroid administration, planned multidisciplinary delivery, and advanced neonatal care were associated with the live birth and survival of all six neonates despite delivery at 28 weeks’ gestation.

This case reinforces the need for careful follicular monitoring, strict cycle cancellation criteria, and comprehensive counseling regarding the risk of multifetal gestation before ovulation induction. When a high-order multiple pregnancy occurs and fetal reduction is declined, management should be individualized at a tertiary care center through coordinated maternal-fetal medicine, obstetric, anesthetic, and neonatal care. Long-term pediatric follow-up remains essential because survival after extreme prematurity does not eliminate the risk of subsequent neurodevelopmental and multisystem complications.
